# Asymmetric competitive suppression between strains of dengue virus

**DOI:** 10.1186/1471-2180-8-28

**Published:** 2008-02-08

**Authors:** Kim M Pepin, Kalli Lambeth, Kathryn A Hanley

**Affiliations:** 1 Department of Biology, New Mexico State University, Las Cruces, NM, 88003, USA

## Abstract

**Background:**

Within-host competition between strains of a vector-borne pathogen can affect strain frequencies in both the host and vector, thereby affecting viral population dynamics. However little is known about inter-strain competition in one of the most genetically diverse and epidemiologically important mosquito-borne RNA virus: dengue virus (DENV). To assess the strength and symmetry of intra-host competition among different strains of DENV, the effect of mixed infection of two DENV serotypes, DENV2 and DENV4, on the replication of each in cultured mosquito cells was tested. The number of infectious particles produced by each DENV strain in mixed infections was compared to that in single infections to determine whether replication of each strain was decreased in the presence of the other strain (i.e., competition). The two DENV strains were added to cells either simultaneously (coinfection) or with a 1 or 6-hour time lag between first and second serotype (superinfection).

**Results:**

DENV2 and DENV4 showed significantly reduced replication in mixed infection relative to single infection treatments. In superinfection treatments, replication was suppressed to a greater extent when the interval between addition of each strain was longer, and when a strain was added second. Additionally, competitive effects were asymmetric: although both strains replicated to similar peak population sizes in single infections, DENV2 was more suppressed than DENV4 in mixed infections. Superinfection treatments yielded significantly lower combined virus titers than coinfection or single infection treatments.

**Conclusion:**

Competition between DENV strains in cultured mosquito cells can cause a significant decrease in peak viral population sizes, which could translate to decreased transmission by the vector. Effects of competition were asymmetric between DENV2 and DENV4, probably reflecting significant variation in the competitive ability of DENV strains in nature. Competition was strongest in superinfection treatments, suggesting that colonization of new DENV strains could be impeded in areas where numerous mosquitoes are infected with endemic DENV strains.

## Background

Infection of a single host by multiple strains of a pathogen may result in competition for host resources [1]. Such intra-host competition is predicted to shape a variety of pathogen traits such as virulence, transmissibility, and resource partitioning, that could affect epidemiology and virus population dynamics [2-9]. However, empirical data demonstrating the action of intra-host competition among pathogens have been scarce, particularly for medically-relevant organisms [10,11]. Among viruses, the magnitude of competition is sensitive to the order and interval of infection by different strains. For example, when two strains of Cydia pomonella granulomavirus infect a codling moth host at the same time (coinfection) replication of both strains is decreased [12], yet coinfection of disparate strains of vaccinia virus in cultured monkey cells does not result in decreased replication [13]. However, in the vaccinia system, when one strain infects four hours after the other (superinfection), replication of the second strain is suppressed, and with a ten-hour lag time between infections, the second strain is unable to replicate at all (superinfection exclusion). Similar patterns, albeit over different timescales, have been observed *in vivo *for Borna disease virus infection of rats [14], LaCrosse virus infection of mosquitoes [15], and bluetongue virus infection of midges [16]. Notably, competitive suppression is not an inevitable outcome of mixed-strain infection. Avirulent strains of murine cytomegalovirus and herpes simplex virus may experience enhanced replication and virulence in mixed-genotype relative to single-genotype infections [17,18]. Thus, while inter-strain interactions appear to significantly impact the population dynamics of viruses, their outcome varies across biological systems. In order to understand and predict the evolutionary epidemiology of medically important viruses that exist as mixed-strain assemblages, it is important to study the interactions within those particular systems.

Dengue viruses (DENV, genus *Flavivirus*, family *Flaviviridae*), the mosquito-borne human pathogens that cause dengue fever, have increased in geographic range, prevalence, and disease severity in recent decades. DENV is currently considered the most significant emerging threat to global public health of any vector-borne virus [19]. Genetic variation within this diverse group of RNA viruses has been categorized as follows: antigenically and genetically distinctserotypes (DEN1–4) are each comprised of numerous distinct genotypes, which are in turn subdivided into multiple sub-groups or types [20]. Genome sequence data and phylogenetic analyses suggest that strain replacement, apparently mediated by competitive displacement, is widespread in DENV epidemiology [20-25]. Most prominently, the Southeast Asian genotype (SA) of DENV2 invaded the Americas in the late twentieth century and has subsequently displaced the endemic American (Am) DENV2 genotype across much of the New World [20,26]. This displacement has had substantial impacts on the epidemiology of DENV because the SA genotype is associated with the most severe manifestations of DENV disease, dengue hemorrhagic fever and dengue shock syndrome, whereas the Am genotype is not [26,27]. At present, there are no licensed vaccines or antiviral therapies available to control DENV, and vector control has proved ineffective at limiting the global spread of the virus [28]. In order to develop new control strategies and effectively deploy existing ones, it will be necessary to gain a better understanding of the factors that shape the ecology and evolution of this virus.

For within-host competition among pathogen strains to happen, multiple strains must occur in the same geographic location, infect the same host, and target the same cells within that host. DENV meets all three of these criteria. The four DENV serotypes co-circulate across most of the geographic range of the virus [29-32] and co-infection of individual humans and vectors by multiple DENV strains occurs in nature [33-36], and even appears to be common in some outbreaks [37]. In the mosquito vector, all four dengue serotypes use the same putative receptor in the mosquito midgut, the initial site of DENV replication [38], and initial infection of the mosquito midgut appears to involve only a few individual cells [39]. The same may be true in human hosts, but the initial stages of DENV infection in humans have proven difficult to characterize. One significant difference between vector and human infection is that the former persists for life whereas the latter is transient because it is cleared by the host immune response. This may expand the opportunities for competition in vectors relative to humans.

To date only one study has explicitly considered the role of competition within the vector on the epidemiological dynamics of wild type DENV strains: among *Aedes aegypti *mosquitoes fed on bloodmeals containing equal quantities of the Am and SA genotype of DENV2, more mosquitoes were infected with the SA genotype [40]. However this study did not include single genotype infections for comparison. Since the Am genotype also has lower infectivity in single infections [41], it is unclear whether the differences in the coinfection study are due to competition within the vector or whether they are simply inherent differences among the genotypes that act independently in mixed infections. While variation between the Am and SA genotypes in their intrinsic ability to infect mosquitoes does appear to contribute to the competitive displacement of the former [40], coinfection studies conducted with the appropriate single infection controls are needed to characterize the role of direct competition among DENV strains infecting a single host.

Data from vaccine research provides the only other experimental evidence suggesting that heterologous DENV strains can interfere with each other's replication during coinfection; studies of the replication of live-attenuated tetravalent vaccine formulations (containing all four serotypes) in humans have shown that the replication of individual serotypes appears to be sensitive to the identity and concentration of coinfecting serotypes [42,43]. Although these data suggest that interference between serotypes can cause decreased replication, it is unclear whether these effects are due to direct competition or whether they are mediated indirectly through the immune system.

To test the hypothesis that competition between dengue virus strains occurs within the mosquito vector, we compared replication of two dengue serotypes (DENV2 and DENV4) in single relative to mixed infections in cultured *Aedes albopictus *mosquito cells (C6/36), a model for replication in one of the primary DENV vectors. Serotypes infected alone (single infections), or together (mixed infections) by either coinfection, where both serotypes were added simultaneously, or by superinfection, where the second serotype was infected either one or six hours after the first. Viral progeny output was measured at three designated time points. Single infections served as controls for interpreting effects of mixed infections. We predicted that if competition occurs: (l) a given serotype would replicate to higher titers in single infections relative to mixed infections, (2) the serotypes, which have similar replication rates in single infections, would be suppressed similarly in mixed infections, and (3) superinfection would affect the degree of competitive suppression such that the longer the interval between the introduction of two serotypes, the more disproportionate the suppression of the serotype infecting second. While predictions 1 and 3 were borne out by our results, we detected intriguing asymmetries in the response of the two serotypes to competition. Our results highlight that intra-host competition could affect transmission of different DENV serotypes and that the degree of suppression depends on time interval between infection of the two serotypes as well as their identity.

## Results

**Figure 1:**
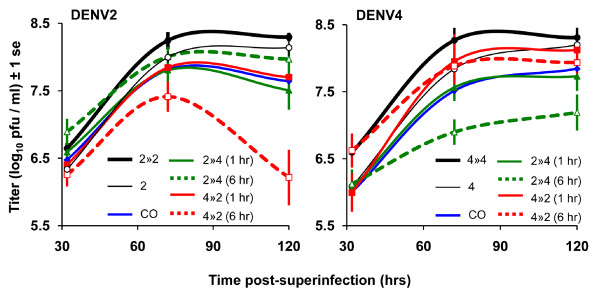
**Titers over time**. Mean titers are plotted against the superinfection interval for DENV2 (left) and DENV4 (right). Bars indicate standard errors of the mean (N = 4). Single-serotype infections are in black, mixed-serotype infection treatments are: coinfections (blue), superinfection of DENV4 after DENV2 (green), and superinfection of DENV2 after DENV4 (red). Treatment abbreviations correspond to the treatments described in Table 1.

The experimental design is described in Table 1. To test whether the replication of a single serotype infecting at a multiplicity of infection (MOI) of 5 was suppressed by an equal dose of the same serotype (e.g. intra-strain competition), we compared the replication of each serotype in a single infection (MOI 5) with its replication after superinfection by the same serotype 1 or 6 hours following the initial infection (MOI 5 + MOI 5 = 10). For example, for DENV2, titers in treatments 1 and 8 (Table 1) were compared with titers in treatments 4 and 11. Each serotype reached significantly higher titers on average at each timepoint when superinfected by the same serotype (Fig. 1, Table 2, left; [Additional File 1]), suggesting that single infections at MOI 5 have not exhausted the potential for host cells to support virus replication, and that serotypes in mixed infections (total MOI 10) had the potential to grow to titers as high as those in their respective single infections of MOI 5 (i.e., they were not limited by density effects).

**Table 1:** Description of Experimental Design.

	**Treatment Number^a^**	**Infection Type**	**Serotypes^b^**	**Superinfection Interval (hrs)**	**Total MOI**
**A**	1	SINGLE	2	0	5
	2	(intraspecific)	4	0	5

	3^c^	CO (inter-)	2 and 4	0	10

	4^c^	SUPER	2 then 2	1	10
	5^c^	(intra-)	4 then 4	1	10
	6	SUPER	2 then 4	1	10
	7	(inter-)	4 then 2	1	10

**B**	8	SINGLE	2	0	5
	9	(intraspecific)	4	0	5

	10^c^	CO (inter-)	2 and 4	0	10

	11^c^	SUPER	2 then 2	6	10
	12^c^	(intra-)	4 then 4	6	10
	13^c^	SUPER	2 then 4	6	10
	14^c^	(inter-)	4 then 2	6	10

^a ^Treatments are numbered; these numbers are referenced throughout the manuscript.

^b ^Numbers refer to the DENV serotypes in the treatment; and = the two serotypes were added simultaneously, then = there was a time lag between the addition of each serotype.

^c ^Treatments used in Figure 3: Single-strain infections = 4, 5, 11 and 12, Coinfections = 3 and 10, and Superinfections = 13 and 14.

**Table 2:** Results of repeated measures ANOVA.

	**Single-strain: MOI 5 vs MOI 10**				**Single-strain MOI 5 vs all mixed-strain (1 level)**				**Single-strain MOI 5 vs each mixed-strain (5 levels)**			
**DENV2**	***DF***	***SS***	***F***	***P > F***	***DF***	***SS***	***F***	***P > F***	***DF***	***SS***	***F***	***P > F***

Treatment	1	0.6	7.3	0.019	1	1.0	2.5	0.12	5	7.4	8.6	0.0001
Subj(Grp)	12	1.0			26	10.2			22	3.8		
Time	2	26.3	198	0.0001	2	27.8	71.8	0.0001	2	23.9	73.1	0.0001
Ti × Tr	2	0.04	0.3	0.74	2	2.1	5.5	0.007	10	5.0	3.1	0.005
Ti × Sub(Grp)	24	1.6			52	10.0			44	7.2		

**DENV4**												

Treatment	1	1.4	15.1	0.002	1	0.8	3.1	0.09	5	4.3	7.1	0.0005
Subj(Grp)	12	1.1			26	6.3			22	2.7		
Time	2	31.1	76.5	0.0001	2	42.3	124	0.0001	2	42.1	132	0.0001
Ti × Tr	2	0.3	0.7	0.5	2	0.6	1.9	0.16	10	2.5	1.6	0.15
Ti × Sub(Grp)	24	4.9			52	8.8			44	7.0		

Ti = Time, Tr = Treatment, Subj = Subject, Grp = group.

To test the prediction that DENV serotypes in mixed infections would replicate to lower titers than DENV serotypes in single infections, titers for each serotype in single infection treatments were compared to those in mixed infection treatments (i.e., with individual mixed treatments pooled as a single effect). DENV2 showed decreased titers in mixed versus single infections, and the magnitude of this effect increased with time (Fig. 1 and Table 2, middle). Interestingly, competition did not appear to affect replication rate during most of the exponential growth phase (compare slopes from 32 to 72 hours in Fig. 1). DENV4, in contrast, showed no significant difference in replication in single versus mixed-strain infections (Fig. 1 and Table 2, middle). However, a separate analysis of the effect of each individual mixed-serotype treatment showed that treatment-type explained a significant amount of variation in DENV4 titers (Fig. 1 and Table 2, right).

**Figure 2:**
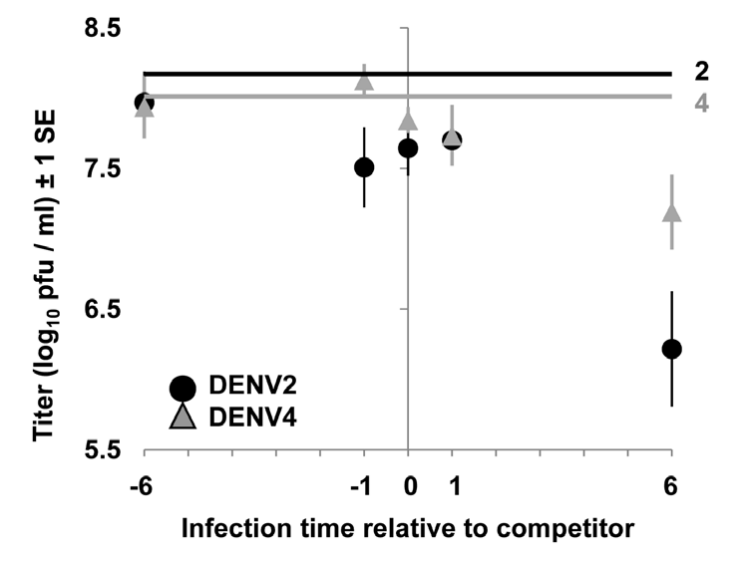
**Effects of the infection time relative to competitor**. Mean titers for DENV2 (circles) and DENV4 (triangles) are plotted against the time that each strain was added to cells relative to the other strain (i.e., the circle at X = -6 indicates the titer of DENV 2 when DENV 2 was infected 6 hours before DENV 4, while the circle at X = 6 indicates the titer of DENV 2 when DENV 2 was infected 6 hours after DENV 4, and X = 0 indicates that both were added at the same time, etc). Titer data are from the final sampling point (120 hours, N = 4 per data point). The titer of each strain in single infection (MOI = 5) is plotted as a straight line for comparison; DENV2 (black) and DENV4 (grey).

To test the prediction that the magnitude of competitive suppression depends on superinfection interval, the titers at 120 hrs post-superinfection for each serotype in mixed infection treatments were regressed on the time that the serotype was infected relative to its competitor. We also included serotype as an effect in this analysis to test the prediction that suppression would be similar for both serotypes. In agreement with prediction, titers for each serotype in mixed infections showed a significant negative relationship with the length of time between their infection and infection by the competitor (Fig. 2, Table 3). That is, the magnitude of competitive suppression for a serotype was stronger, the later that the serotype was added after the other serotype, and this pattern was consistent for both serotypes. The analysis also showed that the intercept of the relationship was higher for DENV4 relative to DENV2, indicating that DENV2 titers were more adversely affected by competition than DENV4.

**Table 3:** Results of ANCOVA.

**Source**	***DF***	***SS***	***F***	***P > F***	**Estimate**	***SE***
**Model**	3	8.3	11.2	0.0001		
**Error**	38	9.4				
**Total**	41	17.7				
**Intercept**					7.6	0.077
**TRC**	1	1.2	4.9	0.033	-0.17	0.077
**DENV**	1	6.2	25.2	0.0001	-0.10	0.021
**TRC × DENV**	1	0.9	3.4	0.0711	-0.04	0.021

TRC = Time in hours that serotype was added Relative to its Competitor; DENV = DENV serotype (2 versus 4)

**Figure 3:**
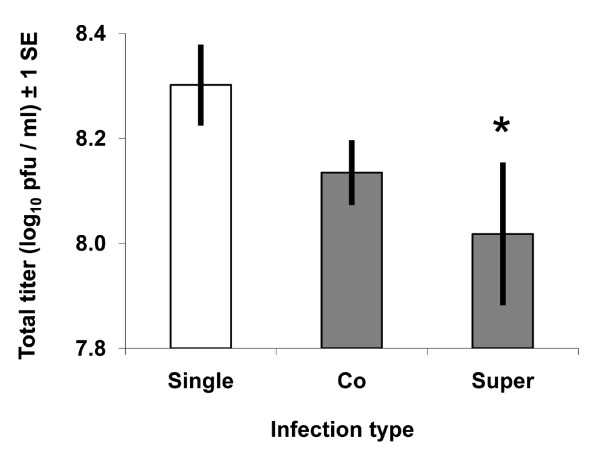
**Effects of mixed infection on total titers**. Mean titers at 120 hours are shown for each type of infection. Bars indicate standard error of the means. White bars represent single-strain infections where each assay received either DENV2 or DENV4 at a total MOI of 10 (4, 5, 11 and 12, Table 1). Thus, the bar is a mean for single-strain infections for all DENV2 and DENV4 replicates averaged together. Grey bars represent mixed-strain infections where each assay received both DENV2 and DENV4 at a MOI of 5 for each. The superinfection category includes both orders of superinfection (2 then 4, 4 then 2; treatments 13 and 14, Table 1), and only the 6-hour superinfection data are included. Coinfections included treatments 3 and 10 from Table 1. Titers of the entire virus population were significantly lower in superinfection treatments than in single-strain infections (indicated by *).

To examine effects of mixed-strain infection on total virus production (i.e., the combined titer for both strains in mixed infections), total titers for coinfection (treatments: 3 and 10, Table 1) and superinfection (treatments: 13 and 14) treatments were each compared to the grand mean of single-strain infection treatments. The single-strain infection category included data for both DENV2 and DENV4 strains, but only the treatments where the total amount of virus added was equivalent to a MOI of 10 (treatments: 4, 5, 11 and 12) since the total MOI in the mixed-strain infection categories was also 10. If mixed infections do not affect total titers, than the average titer of DENV2 and 4 in single-strain infections should equal the sum of DENV2 and 4 in mixed-strain infections, when both treatments are initiated with the same dose of total virus. In coinfections, total titers were similar to those in single-strain infections (*t*_2,19 _= 1.7, *P *< 0.054; Fig. 3), but in superinfections they were in fact lower than in single-strain infections (*t*_2,12 _= 1.8, *P *< 0.048; Fig. 3), indicating that superinfection decreased total viral replication.

## Discussion

To examine whether competition between DENV serotypes can occur, we tested whether high dose infections with two serotypes in cultured mosquito cells affected progeny output for either serotype relative to its output in single-serotype infections. *Aedes albopictus *is one of the major vectors of dengue worldwide [31], and while cultured cells are not a perfect model for infection *in vivo *they offer a tractable system for assessing the potential effects of mixed-serotype infections. We found that replication can indeed be suppressed during mixed infections with two serotypes, and that the magnitude of suppression of a given serotypeis stronger for serotypes infecting cells that already have established infections with a different serotype. This is the first study that quantitatively demonstrates competitive suppression between dengue serotypes. Since DENV isolation studies have suggested that mixed-serotype infections in both hosts and vectors occur, and may even be common in some circumstances, our data indicate that further investigation of the interactions between dengue serotypes is important for understanding dengue epidemiology.

Although competition affected final titers, population growth rate during most of the exponential phase was similar between single infection and mixed infection treatments. Thus, competitive suppression occurred late in infection, once virus populations had approached a plateau in concentration. A similar late-acting effect was observed when a wild type strain of foot-and-mouth disease virus (FMDV) was co-infected with engineered strains carrying 1–3 point mutations in either the capsid, a structural protein, or the polymerase, a non-structural protein responsible for replication of the genome [44]. Wild type virus replication was not suppressed within the first 3–5 hours after introduction to cells, but at later sampling time points the wild type had produced significantly fewer infectious progeny relative to single infection controls. Furthermore, in FMDV suppression of wild type virus replication was not always observed, but rather the outcome depended on the particular mutations carried by the competitor. Replication ability of the competitor mutants was characterized in separate treatments (in single infections), which showed that while some mutants were incapable of producing infectious progeny, others were almost as fit as the wild type. Of the three competitors carrying mutations in the polymerase, only the mutant that produced high levels of polymerase and infectious progeny caused suppression of the wild type. Interestingly, some of the competitors carrying capsid mutations produced large quantities of capsid protein early in infection and actually enhanced the production of infectious particles of wild type early in infection. Taken together, the results suggest that competitive success may depend upon a strain's ability to monopolize its own replication machinery and structural proteins and to acquire those of its competitors late in infection. Our finding that DENV serotypes are suppressed later in infection is consistent with the idea that competition is mediated by the intracellular density of virus genome templates and copies of virus proteins, perhaps through competition for polymerase or capsid proteins [45-48]. Under this hypothesis, a virus strain that experienced high levels of coinfection with other strains, such as a new strain invading an area with an endemic virus population, would benefit from a rapid escalation of RNA replication early in infection. Our results support this notion by the finding that the magnitude of suppression depended upon the superinfection interval. Serotypes that infected earlier had an advantage, whereas those that infected later had the most extreme disadvantage.

The two serotypes of DENV used in this study, which showed similar rates of replication in isolation, nonetheless showed significant variation in competitive ability, suggesting that similar variation may occur in nature. A second implication of this asymmetry is that traits conferring competitive ability in mixed infection may be de-coupled from those that enable high replication rates in single infections. For example, a virus strain may replicate rapidly when it has sole access to homologous viral polymerase but poorly in a mixed population of genomes competing for heterologous polymerases. Importantly, these results underscore that the outcome of within-host competition is not predictable from replication rates in single infections. This finding could also be relevant in the design and development of a tetravalent vaccine, which must contain all four serotypes. If one serotype is better able to usurp polymerase in a coinfected cell, then the production of neutralizing antibodies may be unbalanced, resulting in an ineffective vaccine. In this context, however, the total dose of virus is low enough that opportunity for inter-serotype interference within cells may be limited to peripheral cells co-infected at the injection site.

The fact that total titers were significantly lower in superinfections when compared to single infections of the same MOI shows that interspecific competition was stronger than intraspecific competition, further suggesting that competition-related traits are available to selection in this system. Thus, under the hypothesis that competition for viral proteins mediates suppression, we predict that strains isolated from regions where serotypes frequently coinfect mosquitoes should have the strongest ability to utilize and compete for genetically different polymerase or capsid proteins (i.e., generalists). However, with regard to differences in total titers in single versus mixed infections, there is a slight inconsistency; total titers in coinfections were not significantly lower than single infections while those in superinfections were. Theoretically, in the two treatments the frequencies of cells infected with particular numbers of each serotype would have been similar. The difference is that viruses that superinfect could be at a numerical disadvantage since those that infect first have already begun progeny production and thus could outnumber the second serotype once resources become limiting. Our result that superinfection decreases overall virus production significantly more that coinfection, indicates that competition resulted in disproportionately more severe effects on the total population when one of the strains had a headstart in the infection. This is consistent with our suspicion that the mechanism of competition may be density-dependent (i.e., each serotype is suppressed relatively more when the other serotype is more frequent). Experiments to test this prediction are ongoing.

## Conclusion

Our results demonstrate that competition between serotypes can affect virus titers in mixed infections in mosquito cells, suggesting that competitive suppression could act to decrease transmission. To better understand the role of inter-serotype competition in emergence of dengue, future research should aim to identify predictor variables of suppression, to examine the effects of mixed-serotype infections on replication throughout both stages of the virus life cycle (vector and host), and to quantify these effects in an epidemiological framework. It would also be useful to examine these effects in live mosquitoes. Recent studies of single strain infections in *Aedes aegypti *have highlighted that viruses must replicate in, and disseminate to, several different vector tissues before infecting the salivary glands and disseminating to the saliva for transmission [39,49]. The nature of this pathway and our finding that competitive abilities were uncoupled from performance in single infections highlight that there is potential for interaction effects between serotypes at several stages during vector infection, which could complicate prediction of the effects of mixed serotype infection. Lastly, our results underscore that within-host competition in the mosquito vector may have dramatic effects on both emergence and long-term virus persistence, and these potential effects should be explored in the context of other important factors of dengue virulence such as the host immune system.

## Methods

### Cells and viruses

*Aedes albopictus *epithelial cells (C6/36) [50,51] were maintained in minimal essential media (Invitrogen, Grand Island, NY) supplemented with 10% fetal bovine serum, 2 mM L-glutamine (Invitrogen), 2 mM non-essential amino acids (Invitrogen), and 50 μg/ml gentamycin at 32°C (hereafter termed C6/36 media), 5% CO_2_, and 88% RH. DENV-2-DOO-0372 (DENV2) was originally isolated in Thailand in 1988 from a Type III DHF case and subsequently passaged in C6/36 seven times, with a final titer of 5 × 10^8 ^pfu/ml. DENV-4-Thai85-052 (DENV4) was isolated in Thailand (associated disease unknown) and passaged in C6/36 three times, with a final titer of 4 × 10^8 ^pfu/ml.

### Infections and titering

**Figure 4:**
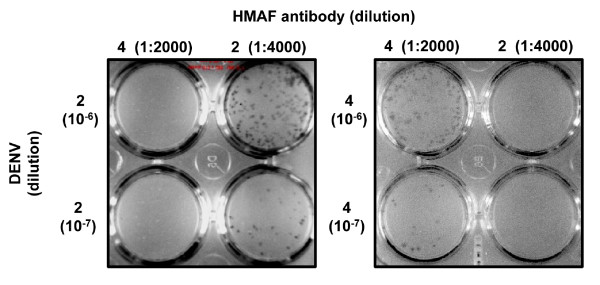
**Specificity of HMAF antibodies**. Stock virus samples containing only DENV2 (left panel) or DENV4 (right panel) were serially diluted and infected on C6/36 cell monolayers at 80–90% confluence in 24-well plates. The two most dilute infections of the ten-fold dilution series are shown. Infections were done in duplicate. Plates were incubated at 32°C, 5% CO_2_, and 88% RH. For staining, DENV2 and DENV4 samples were each incubated with both HMAF antibodies in separate wells (HMAF4: left columns of each panel, HMAF2: right columns of each panel). The antibody dilutions, which were determined to be specific and thus were used throughout the study, are shown.

Virus titers were determined by infecting 80–90% confluent C6/36 cells in 24-well tissue culture treated plates (BD Falcon™, Fisher Scientific) with a tenfold serial dilution of the designated virus in duplicate. Plates were incubated with occasional shaking for 2 hours under conditions for maintenance of C6/36 described above and then overlaid with 1 ml/well of 0.8% methylcellulose in Optimem (Invitrogen) supplemented with 2%FBS, 2 mM L-glutamine and 50 μg/ml gentamycin. Plates were incubated for 5 days at which time plaques were detected by antibody and visualized by immunoperoxidase staining as previously described [52,53] with the following modifications: for one replicate of each duplicate series hyperimmune mouse ascites fluid (HMAF) against DENV4 at a 1: 2000 dilution was used to detect DENV4 and in the other HMAF against DENV2 at a 1: 4000 dilution was used to detect DENV2. HMAF dilutions were optimized to eliminate non-specific staining (Fig 4). Titers were quantified as pfu/ml, where each plaque represents a focus of infection initiated by a single virion and detected by antibody.

To test the impact of mixed infection on replication, designated viruses were added to ~80% confluent monolayers of C6/36 cells in 6-well tissue culture-treated plates (BD Falcon™) at a multiplicity of infection (MOI) of 5 (5 plaque forming units, pfu, per cell). Media was first removed from cell monolayers, cells were washed in 2 ml fresh C6/36 media, and 1 ml of C6/36 media containing the appropriate number of virus particles was added. Cells were then incubated at 32°C for 20 minutes, washed with 2 ml of fresh media, and 3 ml of fresh media was added. For the second infection in the superinfection treatments, media was removed and the virus solution suspended in fresh media was added directly to the monolayer, omitting the wash step that was done in the first infection. Cells were again incubated for 20 minutes, then washed in 2 ml of fresh media, and fed 3 ml of fresh media. All treatments were incubated at 32°C, 5% CO_2_, and 88% humidity; supernatant samples (1 ml) were collected at 32, 72, and 120 hours after the last virus sample was added. This means that in 1-hour superinfection experiments, samples were collected at 33, 73, and 121 hours after the initial infection time, while in 6-hour superinfection experiments, samples were collected at 38, 78, and 126 hours. The 1 ml samples were immediately replaced with fresh media.

### Experimental design

Table 1 summarizes the experimental design. DENV serotypes were infected as single infections (DENV2 or DENV4) or mixed infections (DENV2 and DENV4), the latter including: 1) coinfection in which both strains were added simultaneously, and 2) superinfection in which one strain was added after the other. Two superinfection intervals, 1 hour or 6 hours, and both superinfection orders (DENV2 first or DENV4 first) were used. Each treatment was replicated three times for a total of four independent infections. In one of these replicates, treatments were conducted in duplicate or triplicate in order to assess whether within-treatment effects (e.g., measurement noise from the titering protocol) contributed significant variability to between-treatment effects. A repeated-measures nested ANOVA with treatment as the repeated factor and the within- versus between-replicate effect nested within treatment, showed that there was a significant effect of treatment for both DENV2 and DENV4 (*F*_4,96 _= 8.5, *P *< 0.0001; *F*_4,96 _= 16.4, *P *< 0.0001; respectively) but no significant variation from the nested factor (*F*_5,96 _= 0.4, *P *< 0.86; *F*_5,96 _= 1.1, *P *< 0.4; for DENV2 and 4 respectively). Thus, we concluded that measurement noise from our experimental methods was negligible compared to effects from the imposed treatments. In the replicate where treatments were conducted in multiple-fold, we took the means from the multiple data points to represent the titer of each treatment in that replicate.

### Statistical analyses

All titer data were log-transformed and analyzed by repeated measures factorial ANOVA with assay as the repeated main effect, time as a main effect, and assay × time as an interaction term using StatView (version 5.0.1, SAS Institute Inc.). Since single infection controls and coinfection treatments were not significantly different between the two experiments (1 hour versus 6 hour; [Additional file 1]) these data were pooled as follows: 1+8, 2+9, 4+11, 5+12, and 3+10, where numbers correspond to the treatment labels in Table 1. To test whether serotypes replicated to higher titers when infection was initiated with a greater concentration of virus, single infections of each serotype at MOI 5 were compared to single-serotype superinfections, which had a total MOI of 10 (i.e., 1+8 data versus 4+11 data). To test whether mixed-serotype infection explained a significant amount of variation in titers, the titer of each serotype in single infection treatments of MOI 5 were compared to the titer of that serotype in all mixed infection treatments pooled (e.g., for DENV2: 1+8 versus 3+6+7+10+13+14). Finally, to test the impact of each treatment type on the titer of each serotype, single infection treatments of MOI 5 were compared to each mixed infection treatment considered individually (e.g., for DENV2: 1+8 versus 3+10 versus 6 versus 13 versus 7 versus 14).

The impact of the interval of mixed infections were analyzed by ANCOVA using JMP (Version 5.1, SAS Institute Inc.), where the interval of mixed infection refers to the time that elapsed between addition of first and second serotype (see Table 1 for details). In this analysis, the titer at hour 120 was the dependent variable, the interval of mixed infection was the covariate, and DENV serotype was the discrete factor. To test whether the type of infection treatment influenced the total output of infectious virus (i.e., titers for both serotypes in mixed-strain infections), titers for DENV2 and DENV4 in each mixed infection assay were summed and then log-transformed to generate 'total titer' data. Total titers in coinfections (treatments: 3 and 10, Table 1) and superinfections (treatments: 13 and 14) were each compared to total titers in single-strain infections by *t*-tests using JMP. The single-strain infection category included both strains, but only the treatments where the total amount of virus added was equivalent to a MOI of 10 (treatments: 4, 5, 11 and 12), since the total MOI in the mixed-strain infection categories was also 10.

## Authors' contributions

Experiments conceived by KMP and KAH. Experiments performed by KMP and KL. Data analyzed by KMP. Manuscript written by KMP and KAH.

## Supplementary Material

Additional file 1 Mean titers over time for each treatment. Numerical values depicted in Figure 1. Click here for file
